# The Flow Stress Behavior and Physical-Based Constitutive Model for As-Quenched Al-Zn-Mg-Cu Alloy

**DOI:** 10.3390/ma16144982

**Published:** 2023-07-13

**Authors:** Ruichao Guo, Dandan Liang, Guohua Qin

**Affiliations:** 1 College of Aeronautical Engineering & Shandong Engineering Research Center of Aeronautical Materials and Devices, Binzhou University, Binzhou 256600, China; 17852978839@163.com; 2 School of Aeronautical Manufacturing Engineering, Nanchang Hangkong University, Nanchang 330063, China; qghwzx@126.com

**Keywords:** as-quenched Al-Zn-Mg-Cu alloy, flow stress behavior, constitutive model, precipitation, dislocations

## Abstract

Although heat-treatable Al-Zn-Mg-Cu alloys are widely used in aerospace industries, distortion and cracks exist due to the residual stress during quenching. Understanding the flow stress behavior and numerically modeling the process is the key to predicting the residual stress. This paper investigated the flow stress behavior of the as-quenched 7050 alloy at strain rates from 0.1 s^−1^ to 1 s^−1^, temperatures between 423 K and 723 K, and cooling rates from 0.1 K/s to 10 K/s. The experimental results showed that the strain rate, cooling rate, and temperature have effects on the flow stress value, except for the cooling rates at a temperature of 423 K or 723 K. The kinetics model was used to obtain the precipitate features, i.e., precipitate size and volume fraction. Then, a physical constitutive model based on the evolution of immobile dislocation, solutes, and precipitates was developed. The predicted flow stresses showed good agreement with the experimental data. The findings of this work expand the knowledge on the as-quenched flow behavior of Al-Zn-Mg-Cu alloys, improving the prediction accuracy of residual stress by FEM.

## 1. Introduction

Al-Zn-Mg-Cu (7000 series) alloys have high strength and good toughness and are widely selected as structural component materials for aerospace industries [1]. The structural components are usually manufactured by the initial blanks, which are the aged condition. Aging is one of the heat treatment processes, e.g., solution treatment, quenching, and ageing. In order to obtain enough supersaturated solid solution, the blanks are quenched very quickly. However, a large value of residual stress will result after the quenching process. However, the pre-stretching process can be used to reduce the residual stress. There also exist residual stress, resulting distortion, and cracks for component machining [2,3]. Therefore, it is very important to understand the residual stress distribution.

The precise knowledge of the flow stress behavior for as-quenched materials, which depends on the temperature, strain rate, strain, and microstructures, is the greatest challenge for the prediction of residual stress [4]. An accurate description of the flow behavior in the constitutive model is a difficult task. For aluminum alloys, the constitutive models are mainly divided into three kinds, i.e., phenomenological constitutive model, physical-based constitutive model, and the artificial neural network [5]. The phenomenological constitutive model includes the Arrhenius model, Johnson–Cook (JC) model, Khan–Huang (KH) model, and so on. The physical-based constitutive model includes the dynamic recovery and dynamic recrystallization model, Zerilli–Armstrong (ZA) model, cellular automaton (CA) model, and some other physical-based models.

Many researchers tried to build the constitutive model of as-quenched Al-Zn-Mg-Cu alloys. Ulysse [6,7] built an internal state variable model for the as-quenched 7075 and 7050 alloys. In this model, the state variable was resolved by the phenomenological exponent-type Zener–Hollomon parameter equation. Chobaut et al. [8,9] studied the as-quenched 7449 alloy, and they used a Chaboche-type constitutive model, which can combine the isotropic strain hardening and viscoplastic phenomena. In Chobaut’s work, the threshold stress, which represented the effect of microstructures on the flow behavior, was identified by the inverse method. Reich and Kessler [10] experimentally studied the mechanical properties of undercooled 7020 aluminum alloy. The researchers used a revised empirical Hockett–Sherby hardening law describing the relationship of strain and stress. For the constitutive model of as-quenched 7075 and 7010 alloys, Robinson et al. [11] constructed the Arrhenius equation to generate the flow stress as a function of the temperature and strain rate.

From the above discussions, some models have been built for the as-quenched Al-Zn-Mg-Cu alloys. However, due to their empirical characteristics, the models cannot reflect the influence of the cooling rate on the flow stress behavior. According to the experimental results of [10], cooling rates can strongly influence the flow stress behavior, which is mainly attributed to the precipitation during quenching. The effects of the cooling rates on the flow stress behavior are found not only for the Al-Zn-Mg-Cu alloys, but also for other alloys, e.g., Al-Si alloys [12], Al-Cu-Mg alloys [13,14], and Al-Mg-Si alloys [10].

In the Al-Zn-Mg-Cu alloys, there exist different types of quench-induced precipitation. Starink et al. [15] studied the precipitation phenomenon of Al-Zn-Mg-Cu alloys and found three cooling reactions. In the temperature range of 723 K to 623 K, the S (Al_2_CuMg) phase formed, followed by the η (MgZn_2_) phase forming from 623 K to 523 K and the Zn-Cu phase (deemed as the Y phase in [16]) at a temperature of 523 K to 423 K. In the further studies of [17,18], the results showed that different precipitates had an inconsistent critical cooling rate (CCR). Hence, it can be considered that the quench-induced precipitation is so complicated that the empirical constitutive model used in [6,7,8,9,10,11] cannot identify the impact of different precipitates on the flow stress behavior. Thus, a comprehensive constitutive model, which can fully describe the quench-induced precipitation and the flow stress behavior, is essential for modern residual stress prediction.

In the present work, a constitutive model that can describe the as-quenched flow stress behaviors was built. Firstly, the quench-induced precipitates were predicted by a kinetics model, and two variables (precipitate radius and volume fraction) were considered. Then, a dislocation-based model, considering the forest dislocation, solute element, precipitates, and their interactions, was built. Some isothermal tensile experiments were performed to observe the flow stress behavior.

## 2. Experiments

### 2.1. Material and Experimental Procedure

**Figure 1:**
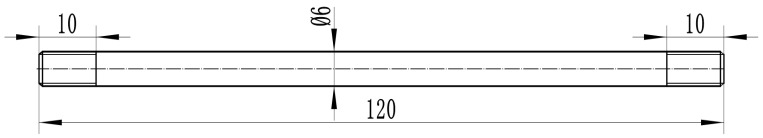
The dimensions of the specimen (units: mm).

The hot-extruded 7050 aluminum alloy was selected in this work. The chemical indices of AA 7050 are listed in Table 1. Based on the standard GB/T 4338-2006 [19], the dimensions of the tensile specimen are shown in Figure 1. Isothermal tensile tests were performed using the Gleeble^®^ 3500 (Dynamic Systems Inc., New York, NY, USA) thermal simulator.

**Figure 2:**
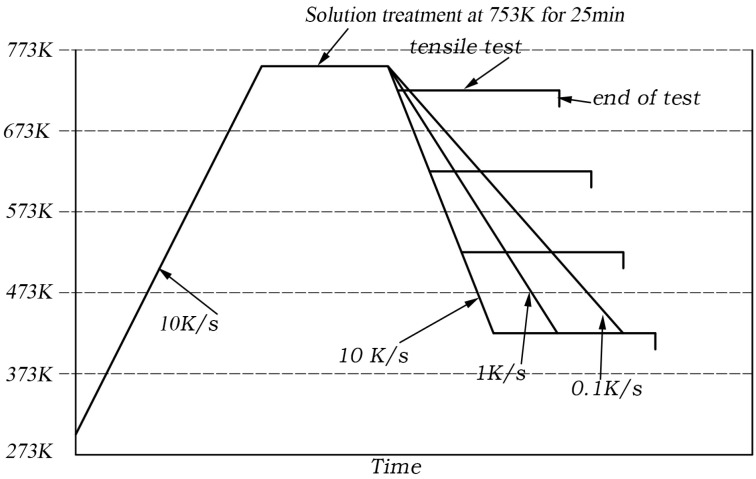
Schematic illustration of heat treatment history.

Firstly, the samples were heated to the solution temperature (753 K) with a rate of 10 K/s. The solution time was 25 min, followed by the quenching process to the desired temperatures with a constant cooling rate. Different parameters such as the temperature, strain rate, and cooling rate were tested, as shown in Table 2. A schematic illustration of the heat treatment history is shown in Figure 2. Continuous cooling diagrams (CCDs) were used to choose the cooling rate parameters. According to [20], the CCRs for the high-, medium-, and low-temperature reactions were 10, 100, and 300 K/s, respectively. Since the temperatures used in this work covered all temperature reactions, cooling rates of 0.1, 1 and 10 K/s were investigated. The isothermal tensile tests were ceased when the strain was 0.2, and the samples were cooled to room temperature immediately.

### 2.2. Experimental Results of Tensile Test

**Figure 3:**
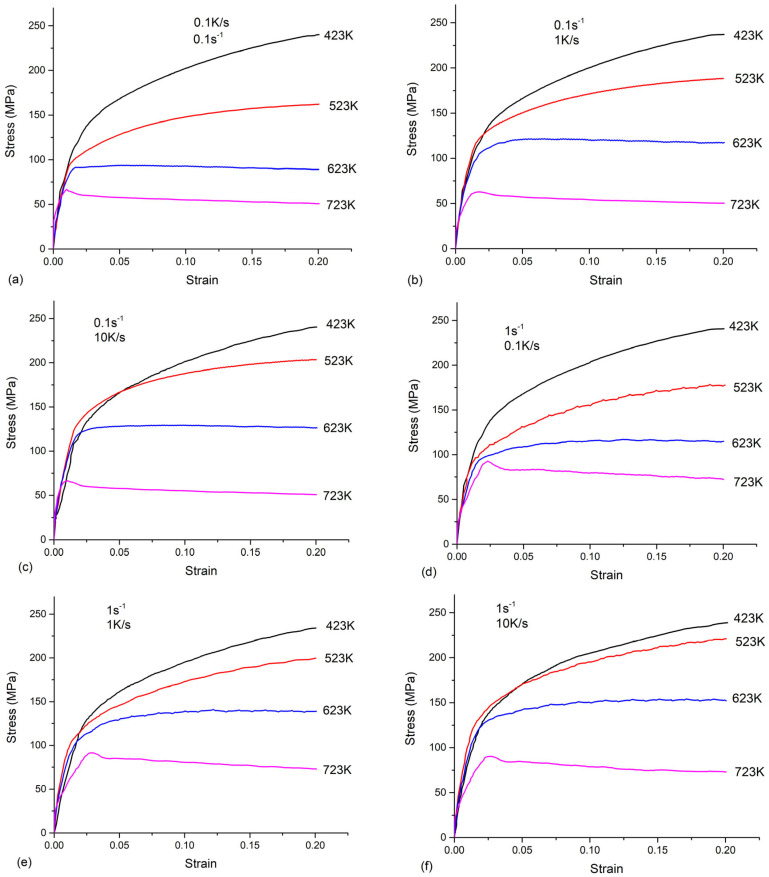
Stress-strain curves of as-quenched AA 7050 at different cooling rates and strain rates.

The strain–stress curves of the as-quenched AA 7050 for different strain rates, cooling rates, and temperatures are shown in Figure 3. It can be seen that the strain rates and temperatures had a strong effect on the flow stress. The flow stress increased with the decreased temperatures or increased strain rates. The maximum flow stress value was found at a temperature of 423 K, a strain rate of 0.1 s^−1^, and a cooling rate of 0.1 K/s. When the temperature was 423 K or 523 K, the stress increased with the strain, which showed a strain-hardening effect. When the temperature was 623 K or 723 K, the flow stress remained steady after reaching the peak stress. This phenomenon occurred when the dynamic softening and work hardening reached equilibrium, i.e., dynamic recovery. The observations were in good agreement with earlier reports [21,22] about AA 7050.

**Figure 4:**
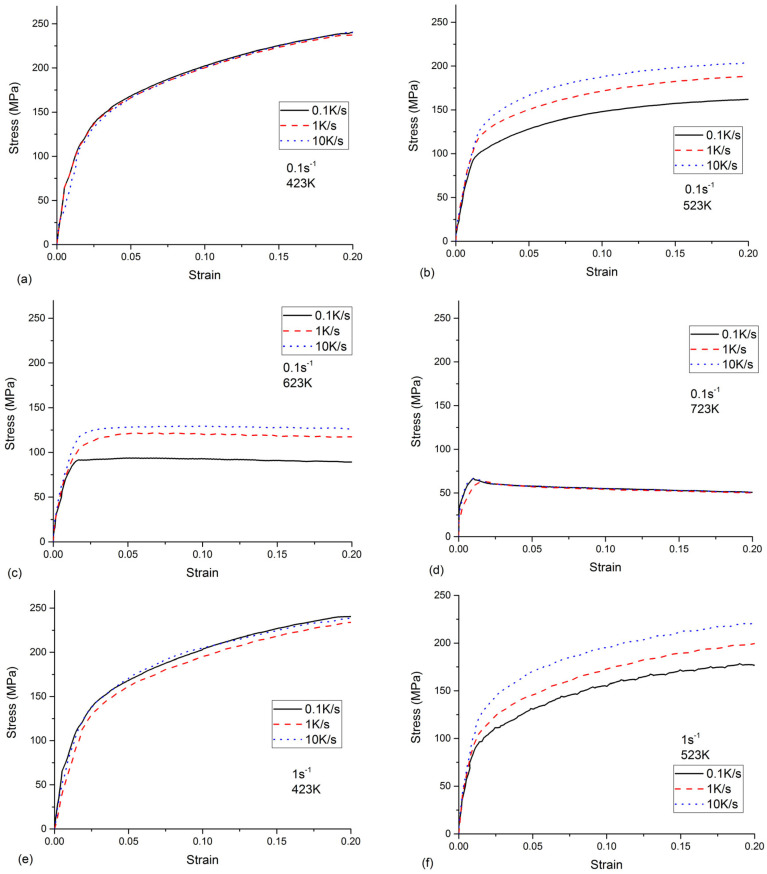

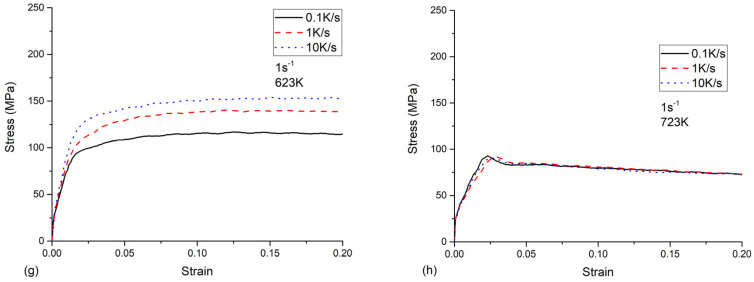
The effects of cooling rates on the flow stress of as-quenched AA 7050.

The effects of the cooling rate on the flow stress at different strain rates and temperatures are shown in Figure 4. When the temperature was 423 K or 723 K, the flow stresses remained constant for different cooling rates. According to [20], the amount of quench-induced precipitates at a high temperature (723 K) or a low temperature (423 K) is minor. Therefore, these quench-induced precipitates had a negligible effect on the strengthening. However, the flow stresses strongly depend on the cooling rates at temperatures of 523 K or 623 K. The flow stress increased with the increasing cooling rates. Based on the experimental results of the step-quench [15], the quench-induced precipitates were the coarse η (MgZn_2_) phase at a temperature of 523 K or 623 K, which is the most-detrimental precipitation. The coarse precipitates led to a lowered solute concentration. Thus, the cumulated effects of the quench-induced coarse precipitates and the loss of solute elements weakened the strengthening. This observed phenomenon was similar to the finding in [10]. Due the non-negligible effects of the cooling rate on the flow stress for temperatures of 523 K and 623 K, the flow behavior cannot be described by the phenomenological model, e.g., the Arrhenius model [21]. Thus, considering the quench-induced precipitates, a physical-based constitutive model is built in the following section.

## 3. Physical-Based Constitutive Model

### 3.1. The Kinetics Model of Precipitation

The precipitation kinetics model was based on the KWN model [23], in which the nucleation, growth, and coarsening processes can be considered. In this model, the input parameters included the temperature, time, and initial concentration of the elements. The outputs of the model were the radius and volume fraction of the precipitates. Starink et al. [15] observed the quench-induced precipitates for 7050 alloys by TEM. The experimental results showed that the precipitates were irregularly shaped. Since the calculated parameter was the mean radius, all the irregularly shaped precipitates were deemed as spherically shaped precipitates and only one kind of quench-induced precipitate, i.e., MgZn_2_, was considered.

The precipitate nucleation rate from the supersaturated solution can be expressed as [24]:

(1) $$\frac{dN}{dt}=N_{0}Z_{f}\beta^{*}\exp\left(-\frac{\Delta G^{*}}{kT}\right)\exp\left(-\frac{\tau_{in}}{t}\right)$$

where *N*_0_ is the nucleation site number per unit volume, $\beta^{*}$ is the atomic attachment rate, *Z_f_* is the Zeldovich factor, $\Delta G^{*}$ is the nucleation activation energy barrier, k is the Boltzmann constant, and $\tau_{\mathrm{in}}$ is the nucleation incubation time, where $\tau_{\mathrm{in}}=2/\pi \beta^{*}Z_{f}^{2}$.

The nucleation activation energy barrier $\Delta G^{*}$ can be provided as:

(2) $$\Delta G^{*}=\frac{16\pi \gamma^{3}}{3\Delta g_{v}^{2}}$$

where γ is the interfacial energy; $\Delta g_{v}$ is the chemical driving force for nucleation, defined as:

(3) $$\Delta g_{v}=-\frac{RT}{V_{molar}^{pre}}\left[X^{pre}\ln\left(\frac{X}{X^{eq}}\right)+\left(1-X^{pre}\right)\ln\left(\frac{1-X}{1-X^{eq}}\right)\right]$$

where *R* is the gas constant, $V_{molar}^{pre}$ is the molar volume of the precipitates, *X* is the present concentration of the solute in the matrix, $X^{pre}$ is the mole fraction of the solute elements in the precipitates (assumed to be Mg), and $X^{eq}$ is the equilibrium solute concentration in the matrix and is calculated by considering the Gibbs–Thomson effect [25]:

(4) $$X^{eq}={\left[\exp\left(-2\right)\exp\left(-\frac{\Delta H}{RT}\right)\right]}^{0.5}$$

where ΔH is the formation enthalpy.

The atomic attachment rate $\beta^{*}$ has a relationship with *X*, the diffusion coefficient for solute *D*, and the critical nucleus size $r_{cp}^{*}$, shown as [24]:

(5) $$\beta^{*}=\frac{4\pi r_{cp}^{*2}DX}{a^{4}}$$

where *a* is the lattice parameter and $r_{cp}^{*}$ is the critical nucleus size, which can be calculated by:

(6) $$r_{cp}^{*}=-\frac{2\gamma }{\Delta g_{v}}$$

The diffusion coefficient for solute *D* is expressed as [26]:

(7) $$D=D_{0}\exp\left(-\frac{Q_{d}}{RT}\right)$$

where *D*_0_ is the material coefficient of the solute and *Q_d_* is the diffusion activation energy.

The diffusion-controlled growth rate of the precipitates is obtained through the balance of the precipitates and particles:

(8) $$\frac{dr_{p}^{*}}{dt}=\frac{D}{r_{p}^{*}}\left(\frac{X-X\left(r_{p}^{*}\right)}{X^{eq}-X\left(r_{p}^{*}\right)}\right)$$

where $r_{p}^{*}$ is the nucleus size, expressed as $r_{p}^{*}=r_{cp}^{*}+0.5\sqrt{kT/\pi \gamma }$), and $X\left(r_{p}^{*}\right)$ is the solute concentration at the precipitate/matrix interface, expressed as [27,28]:

(9) $$X\left(r_{p}^{*}\right)=X^{eq}\exp\left(\frac{2\gamma V_{molar}^{pre}}{X^{pre}RTr_{p}^{*}}\right)$$

The solute element composition can be mass balanced by

(10) $$X=\frac{X_{0}-f_{v}X^{pre}}{1-f_{v}}$$

where $X_{0}$ is the initial solute molar fraction and *f*_v_ is the volume fraction of the precipitate. The precipitate radius can be discretized into several size classes. The kinetic model is calculated for each size class to obtain the final volume fraction, expressed as:

(11) $$f_{v}=\sum_{i=1}^{n}\frac{4\pi r_{p}^{*3}}{3}N_{i}\left(r_{p}^{*}\right)$$

### 3.2. Dislocation Density Flow Stress Model

The as-quenched flow stress behavior resulted from the dislocation movement. The barriers of the movement were immobile dislocations, solutes, precipitates, etc. The resistance, i.e., the shear stress, can be calculated by the additive form:

(12) $$\tau =\tau_{G}+\tau_{ss}+\tau_{p}$$

where τ is the shear stress, $\tau_{G}$ is the athermal stress caused by immobile dislocations, $\tau_{ss}$ is the shear stress caused by the solid solution, and $\tau_{p}$ is the shear stress caused by the precipitates.

The athermal stress $\tau_{G}$ is a long-range term, which is temperature-independent, shown as

(13) $$\tau_{G}=\alpha Gb\sqrt{\rho_{i}}$$

where α is the proportionality factor, b is Burger’s vector, $\rho_{i}$ is the immobile dislocation density, and G is the shear modulus, which has a relationship with the temperature [22]:

(14) $$G\left(T\right)=\mu_{0}\left[1+\frac{T_{M}}{\mu_{0}}\frac{d\mu }{dT}\frac{\left(T-300\right)}{T_{M}}\right]$$

where $\mu_{0}$ is the shear modulus at a temperature of 300 K ($2.54\times 10^{4}$ MPa), *T*_M_ is the melting temperature of aluminum (933 K), and $\frac{T_{M}}{\mu_{0}}\frac{d\mu }{dT}$ is the parameter, which equals −0.5.

During the deformation, the change of the dislocation density has two parts, i.e., the athermal storage and dislocation annihilation:

(15) $$\overset{•}{\rho_{i}}=k_{1}\sqrt{\rho_{i}}-k_{2}\rho_{i}$$

where $k_{1}$ is a material constant describing the dislocation storage rate and *k*_2_ is a parameter associated with the dynamic recovery. Since the dislocation annihilation is a thermally activated process, the parameter *k*_2_ is a function of temperature, expressed as

(16) $$k_{2}=k_{20}+C\exp(-\frac{Q_{vm}}{3RT}){\overset{•}{\varepsilon }}^{-1/3}$$

where $k_{20}$ and C are material constants and $Q_{vm}$ is the vacancy migration activation energy.

Assuming the dislocation density is $\rho_{i0}$ when the plastic strain is 0, the dislocation density in Equation (15) can be calculated by

(17) $$\sqrt{\rho_{i}}=\sqrt{\rho_{i0}}+\left(\frac{k_{1}}{k_{2}}-\sqrt{\rho_{i0}}\right)\left(1-\exp\left(-\frac{1}{2}k_{2}\varepsilon_{p}\right)\right)$$

The solute strengthening is a short-range issue. The dislocation glide overcomes the barrier of the solutes with the assistance of thermal activation. The contribution of $\tau_{ss}$ can be calculated as [29]

(18) $$\tau_{ss}=\tau_{0}{\left[1-{\left(\frac{kT}{\Delta f_{0}Gb^{3}}\ln\frac{\overset{•}{\varepsilon_{0}}}{\overset{•}{\varepsilon }}\right)}^{1/q}\right]}^{1/p}$$

where $\tau_{0}$ is the shear stress at zero temperature, $\Delta f_{0}Gb^{3}$ is the total energy barrier for the dislocation movement by thermal activation and $\overset{•}{\varepsilon_{0}}$ is a constant.

The precipitates act as geometric barriers to the dislocation movement. The dislocation may bypass or shear the precipitates, depending on the precipitates’ characteristics. The earlier findings [30] for the Al-Zn-Mg-Cu alloy showed that the transition radius is about 3 nm. According to the TEM observation in [15], the diameter of MgZn_2_ precipitates is about 100 nm. Thus, in the present work, the radius of the quench-induced precipitates (MgZn_2_ phase) was larger than the transition radius. The obstacle strength required to move dislocations to bypass precipitates is expressed as Orowan bowing [31]:

(19) $$\tau_{p}=\sqrt{\frac{6}{\pi }}\varphi \beta Gb\frac{\sqrt{f_{v}}}{r_{p}^{*}}$$

where β is a dislocation line tension parameter and φ is an efficiency factor considering the influence of the Orowan loop stability.

Finally, the shear stress can be converted to normal stress by the equation:

(20) σ=Mτ

where *M* is the Taylor factor, which has a value of 3.06 [32].

### 3.3. Summary of the Precipitation Kinetics and Constitutive Models

Using the precipitation kinetics model, the precipitates’ characteristics, i.e., the radius and the volume fraction, can be obtained. The solution of the kinetics model was performed numerically using a Lagrange-like approach. Table 3 gives the data for the precipitation kinetics model’s calculation.

Some known parameters used for the constitutive model are listed in Table 4. The rest parameters have the domains (Table 4) and can be calculated by the optimization technique using the experimental data. In the present work, the genetic algorithm (GA) method was selected using a Matlab-based toolbox (2014a). The objective function (*f_x_*) is defined as minimizing the sum of the squared errors between the experimental data and the calculated data, expressed as

(21) $$f\left(x\right)=\sum_{j=1}^{n}\sum_{i=1}^{m}{\left(\sigma_{ij}^{c}-\sigma_{ij}^{e}\right)}^{2}$$

where *n* is the strain rate, *m* is the temperature, $\sigma_{ij}^{c}$ is the calculated stress, and $\sigma_{ij}^{e}$ is the experimental stress.

Since the precipitate hardening is negligible, there are two parts ($\tau_{\mathrm{ss}}$ and $\tau_{G}$) at a temperature of 423 K or 723 K. In general, the constitutive model for the flow stress prediction of the as-quenched Al-Zn-Mg-Cu alloy is expressed as follows:

(22) $$\begin{array}{l} \tau =\tau_{G}+\tau_{\mathrm{ss}}+\tau_{p}\\ \tau_{G}=\alpha Gb\sqrt{\rho_{i}}\\ \tau_{ss}=\tau_{0}{\left[1-{\left(\frac{kT}{\Delta f_{0}Gb^{3}}\ln\frac{\overset{•}{\varepsilon_{0}}}{\overset{•}{\varepsilon }}\right)}^{1/q}\right]}^{1/p}\\ \tau_{p}=\sqrt{\frac{6}{\pi }}\varphi \beta Gb\frac{\sqrt{f_{v}}}{r_{p}^{*}}\;\;\;\;T=523K\;or\;623K\\ \overset{•}{\rho_{i}}=k_{1}\sqrt{\rho_{i}}-k_{2}\rho_{i}\\ \sigma =M\tau \end{array}$$

## 4. Model Prediction Results and Discussions

**Figure 5:**
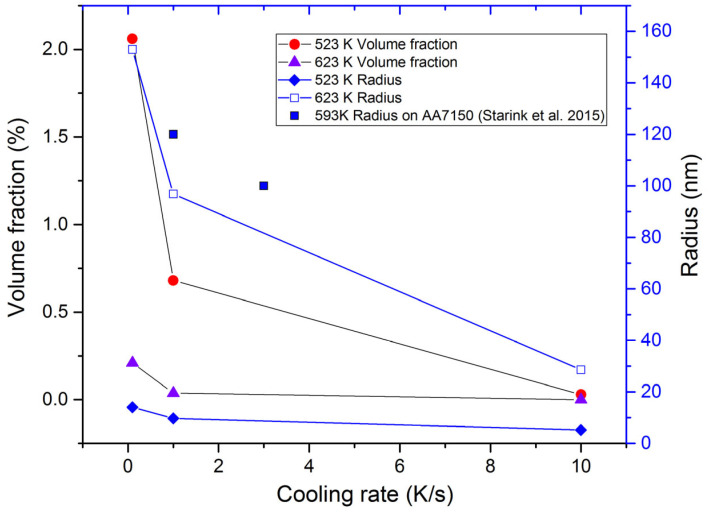
The predicted volume fraction and radius varied with the cooling rate at different temperatures. Literature data are included as points and referenced [15].

The calculated evolution of the volume fraction and radius varied with the cooling rate at different temperatures, as shown in Figure 5. Starink et al. [15] gave the step-quench experiments on AA 7150, the samples being cooled at 3 K/s and 1 K/s to 593 K. To compare the experimental results, the literature data are also included in Figure 5. According to Straink’s results, the radius of MgZn_2_ precipitates is about 100 nm (3 K/s) and 120 nm (1 K/s), respectively. In the present precipitation prediction, both the volume fraction and the radius of the precipitates decreased with the increasing cooling rate. The variation trend was in agreement with the radius reported in the literature [15]. The experimental results for the radius were larger than the predicted radius. One reason may be the irregularly shaped precipitates by the TEM analysis. In the present work, the precipitates were assumed as spherically shaped, resulting in a small calculation. Another reason is the activation energy for diffusion. The η (MgZn_2_) precipitates nucleated on dispersoids, grain boundaries, and so on. The activation energy barrier was inconsistent with different nucleation locations. An easy nucleation position requires a lower activation energy, leading to larger precipitates.

**Figure 6:**
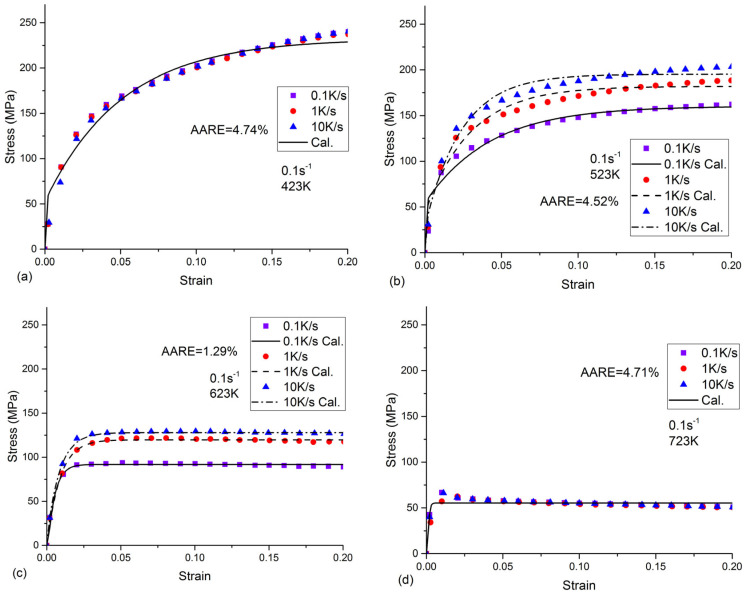

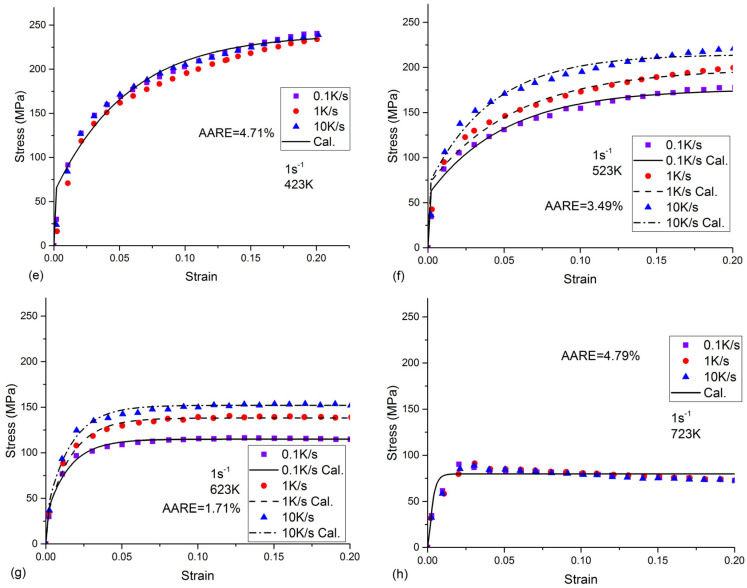
Comparison of experimental (scatter points) and calculated (curves) strain-stress relationships at different temperatures, strain rates, and cooling rates.

The experimental and calculated strain–stress data are plotted in Figure 6. The curve in this figure corresponds to the calculated curve obtained by the physical constitutive model with optimized parameters using the GA-based optimization method. The scatter points denote measurements.

The results in Figure 6 show that there was good agreement between the experimental results and the physical constitutive model predictions. To evaluate the accuracy of the model, the average absolute relative error (AARE) is defined as the following equation [39]:

(23) $$\mathrm{AARE}=\frac{1}{N}\sum_{i=1}^{N}\left|\frac{\sigma_{cal}-\sigma_{exp}}{\sigma_{exp}}\right|\times 100\%$$

where *N* is the predicted flow stress at different strain rates and cooling rates, $\sigma_{cal}$ is the calculated flow stress, and $\sigma_{exp}$ is the measured flow stress.

The AARE based on Equation (23) is also demonstrated in Figure 6. As can be seen, the AAREs for different strain rates, cooling rates, and temperatures were relatively low. Thus, the developed model can successfully be used to describe the flow stress behavior of the as-quenched Al-Zn-Mg-Cu alloy with variable conditions.

## 5. Conclusions

The flow stress behavior of the as-quenched AA 7050 during uniaxial loading was investigated for different cooling conditions. The precipitate features obtained from the kinetics model were used to calculate the resistance in plastic deformation. The flow stress caused by the immobile dislocation was deemed as athermal stress, and the solutes were considered as thermal stress. Three components, i.e., immobile dislocations, solutes, and precipitates, were considered in physical-based constitutive model. The following conclusions were drawn from the present study:

(1) All the tested parameters, i.e., cooling rates, strain rates, and temperatures, can have effects on the flow stress behavior of the as-quenched AA7050 alloy. The values of the flow stress increased with the increased strain rate and the decreased temperature. When the temperature was 523 K or 623 K, the flow stress values increased with the increasing cooling rate. The flow stress values increased with the increasing cooling rate at a temperature of 523 K or 623 K, compared to the negligible effects on flow stress behavior at a temperature of 423 K or 723 K.
(2) In the present precipitation prediction, both the volume fraction and the radius of the precipitates decreased with the increasing cooling rate. The physical-based constitutive model was applied to predict the flow stress behavior in the isothermal tensile test of the as-quenched AA 7050 alloy. The AARE between the calculated and the experimental flow stress was about 4%. The present constitutive model, which considered the effects of the precipitates, can be used to describe the flow stress behavior of other series of as-quenched aluminum alloys.

## Figures and Tables

**Table 1:** Chemical indices in AA 7050.

Chemistry	Zn	Cu	Mg	Fe	Si	Mn	Ti	Others	Al
wt.%	6.2	2.1	2.2	0.05	0.02	0.66	0.02	0.05	Bal.

**Table 2:** The test parameters in the present work.

Aluminum Alloy	Temperature (K)	Cooling Rate (K/s)	Strain Rate ($s^{-1}$)
7050	723, 623, 523, 423	0.1, 1, 10	0.1, 1

**Table 3:** Summary of precipitation kinetics model parameters.

Parameters	Meaning	Value	Source
*a*	Lattice parameter	4.04 × 10^−10^ m	[33]
γ	Interfacial energy	0.3 J/m^2^	[26]
*k*	Boltzmann’s constant	1.38 × 10^−23^ J/K	-
*R*	Gas constant	8.314 J/(mol·K)	-
$V_{molar}^{pre}$	Molar volume of MgZn_2_	3.025 × 10^−5^ m^3^/mol	[26]
$N_{0}$	Nucleation site number per unit volume	1 × 10^28^ m^−3^	[33]
$D_{0}$	Material coefficient of solute	1.49 × 10^−5^ m^2^/s	[34]
$Q_{d}$	Activation energy for diffusion	120.5 kJ/mol	[34]
*Z_f_*	Zeldovich factor	1/20	[31]
$X^{pre}$	Molar fraction of solute Mg in MgZn_2_	0.33 (at.%)	[35]
ΔH	Formation enthalpy	75 kJ/mol	[26]
$X_{0}$	Initial solute concentration of Mg in the matrix	0.0376 (at.%)	-

**Table 4:** Known or domains of parameters for the constitutive model.

Parameters	Meaning	Value or Domain	Source
*M*	Taylor factor	3.06	[32]
*b*	Burger’s vector	2.84 × 10^−10^ m	-
β	Dislocation line tension parameter	0.43	[31]
$Q_{vm}$	Vacancy migration activation energy	68 kJ/mol	[33]
$\overset{•}{\varepsilon_{0}}$	Reference strain rate	$10^{6}\;s^{-1}$	[29]
$\rho_{i0}$	Initial immobile dislocation density	$10^{12}\;m^{-2}$	[36]
α	Proportionality factor	0.2≤α≤0.8	[37]
$\Delta f_{0}$	Dimensionless factor	$0<\Delta f_{0}\leq 2$	[38]
$\tau_{0}$	Shear stress at zero temperature	$50\leq \tau_{0}\leq 2000$	-
*k*_1_	Material constant	$10^{6}\leq k_{1}\leq 10^{10}$	[36]
$k_{20}$	Material constant	$5\leq k_{0}\leq 50$	[36]
*C*	Material constant	$10^{2}\leq C\leq 10^{5}$	[36]
*q*	Material constant	1≤q≤2	-
*p*	Material constant	0<p<1	-
φ	Efficiency factor	0<φ<1	-

## Data Availability

No new data were created nor analyzed in this study. Data sharing is not applicable to this article.
